# Simulating Mars Drilling Mission for Searching for Life: *Ground-Truthing* Lipids and Other Complex Microbial Biomarkers in the Iron-Sulfur Rich Río Tinto Analog

**DOI:** 10.1089/ast.2019.2101

**Published:** 2020-09-15

**Authors:** Laura Sánchez-García, Miguel A. Fernández-Martínez, Mercedes Moreno-Paz, Daniel Carrizo, Miriam García-Villadangos, Juan M. Manchado, Carol R. Stoker, Brian Glass, Victor Parro

**Affiliations:** ^1^ Centro de Astrobiología (CSIC-INTA), Madrid, Spain.; ^2^ NASA Ames Research Center, Moffett Field, California.

**Keywords:** Lipid biomarkers, LDChip, *IceBreaker* prototype drill, Life detection, Planetary exploration

## Abstract

Sulfate and iron oxide deposits in Río Tinto (Southwestern Spain) are a terrestrial analog of early martian hematite-rich regions. Understanding the distribution and drivers of microbial life in iron-rich environments can give critical clues on how to search for biosignatures on Mars. We simulated a robotic drilling mission searching for signs of life in the martian subsurface, by using a 1m-class planetary prototype drill mounted on a full-scale mockup of NASA's Phoenix and InSight lander platforms. We demonstrated fully automated and aseptic drilling on iron and sulfur rich sediments at the Río Tinto riverbanks, and sample transfer and delivery to sterile containers and analytical instruments. As a ground-truth study, samples were analyzed in the field with the life detector chip immunoassay for searching microbial markers, and then in the laboratory with X-ray diffraction to determine mineralogy, gas chromatography/mass spectrometry for lipid composition, isotope-ratio mass spectrometry for isotopic ratios, and 16S/18S rRNA genes sequencing for biodiversity. A ubiquitous presence of microbial biomarkers distributed along the 1m-depth subsurface was influenced by the local mineralogy and geochemistry. The spatial heterogeneity of abiotic variables at local scale highlights the importance of considering drill replicates in future martian drilling missions. The multi-analytical approach provided proof of concept that molecular biomarkers varying in compositional nature, preservation potential, and taxonomic specificity can be recovered from shallow drilling on iron-rich Mars analogues by using an automated life-detection lander prototype, such as the one proposed for NASA's *IceBreaker* mission proposal.

## 1. Introduction

Searching for organics, molecular biomarkers, and other signs of past or extant life in Mars is one of the key objectives for present and future planetary exploration. Despite the current inhospitable conditions of the martian surface (dry, cold, and exposed to high levels of ionizing radiation), the planet may have been habitable for microbial life early in its history, with abundant sources of energy, carbon, nutrients, and shelter (Cabrol, 2018). Findings on the existence of water (liquid in the past, frozen in present) on Mars (Mellon *et al.*, 2009; Martín-Torres *et al.*, 2015; Villanueva *et al.*, 2015) raised the probabilities of finding signs of life on that planet and motivating future exploration missions.

The Mars *IceBreaker* Life mission concept (McKay *et al.*, 2013) is conceived to return to the northern Mars polar latitudes first visited by the Phoenix lander in 2008 to drill and sample the martian permafrost. The mission plans to go through the hard ice-cemented layers that the Phoenix mission (*i.e*., the first astrobiological mission devoted to sample ground ice) encountered, with the aim of acquiring material to search for organic molecules and specific unequivocal molecular biomarkers down to 1m depth.

Given the excessive costs and risks associated with full Mars missions, maturing the technology, procedures, and the analytical techniques in terrestrial analogs is mandatory. Simulation campaigns enable troubleshooting and tune the whole process of sampling, material delivery and distribution, as well as *in situ* analysis. Extreme terrestrial environments analogous to Mars offer great potential for the simulation campaigns, as well as for making progress in understanding how life may have adapted, spread, and left its fingerprints in the apparently inhospitable martian conditions.

The Río Tinto area (Southwestern Spain) provides a series of extreme settings considered as geochemical and mineralogical analogs of certain regions in Mars. Particularly useful are the similarities between the Río Tinto sulfide bioleaching products and the vast sulfate and iron oxide deposits detected in the martian *Meridiani Planum* rocks (Fernández-Remolar *et al.*, 2005). The extremophilic microorganisms inhabiting the Río Tinto environment generate biosignatures that can be preserved in remarkable detail within iron sulfate (jarosite), iron oxyhydroxide (goethite), and iron oxide (hematite) minerals (Preston *et al.*, 2011). The detection by NASA's Opportunity rover of jarosite and hematite on *Meridiani Planum* (Klingelhöfer *et al.*, 2005) suggested the possibility of preserving signs of present or past life (if any) on this or other iron-rich environments on Mars.

Searching for molecular biomarkers in the northern martian terrains will require sample acquisition below the desiccated and irradiated surface, and through hard ice-cemented layers. Mars exploration drills must work dry (without lubricants), blind (no previous local or regional seismic or other surveys), light (very low downward force), and given the lightspeed transmission delays to Mars, automatic (no direct control from Earth). A decade of evolutionary development by NASA of integrated automated drilling and sample handling, at analog sites and in test chambers, has made it possible to go deeper through hard rocks and ice layers (Glass *et al.*, 2008, 2014).

The NASA-funded Life-detection Mars Analog Project (LMAP) over 3 years (2014–2017) created a field brassboard similar in some respects to the proposed *Icebreaker* mission lander (McKay *et al.*, 2013), including its full-size InSight/Phoenix-derived lander platform, the Honeybee Robotics Trident drill, a sample transfer arm/scoop, and the Signs of Life Detector (SOLID) immunoassay instrument (Parro *et al.*, 2011). In February 2017, the Trident drill was tested at a high-fidelity analog site in the Atacama Desert (Chile) as part of the Atacama Rover Astrobiology Drilling System (ARADS) field experiment, where fully automated drilling and sample transfer were successfully demonstrated.

A new testing campaign was then conceived for both maturing the technical performance of the automated Trident drill and examining the bioanalytical value of the acquired samples, at the Río Tinto analog site. Samples (cuttings) obtained with the drill were automatically collected from an acidic, evaporitic site at Río Tinto and autonomously transferred to containers for offline detection of molecular biomarkers. A complete bioanalytical test, including lipid biomarkers, 16S/18S rRNA genes sequencing, and microarray immunoassays with life detector chip (LDChip; the core sensor of the SOLID instrument), was applied to subsamples collected at 20 cm-depth intervals up to 1 m depth.

As a “ground-truth,” the same multi-analytical detection approach was applied to core samples manually collected in parallel with a commercial vibro-corer drill down to 2-m depth. The biomarkers detected on the automatically acquired Trident drill samples (cuttings) were compared with those collected by the manual coring drill to validate the analytical functionality of the *IceBreaker* sampler. Simulation experiments involving organic-containing natural samples are highly valuable for mission design, as they contribute to constrain how many samples should be analyzed, what is the heterogeneity and how to deal with it, or what is the minimal information to define the mission threshold and baseline to be considered successful. This biogeochemical study constitutes a first approach for the field work and bioanalytical detection accomplished on an ideally and successfully funded Mars *IceBreaker*-life mission.

## 2. Materials and Methods

### 2.1. Study area

The Río Tinto basin hosts an acidic aqueous system driven by iron hydrochemistry, where highly acidic waters precipitate iron sulfates and oxides similar to those found in *Meridiani* outcrop rocks on Mars (Fernández-Remolar *et al.*, 2005). Río Tinto sources from springs in *Peña de Hierro*, in the core of the Iberian Pyritic Belt (IPB), and the characteristic acidic pH of its reddish waters are the result of the activity of an underground bioreactor that obtains its energy from the massive sulfide deposits (Amils *et al.*, 2014). Microbial metabolism's fingerprints from chemolithotrophic microorganisms thriving in the high concentration of the IPB iron sulfides may be preserved within iron minerals (*e.g*., jarosite, goethite, and hematite) that are abundantly found in the Río Tinto basin (Fernández-Remolar *et al.*, 2004; Fernández-Remolar and Knoll, 2005; Parro *et al.*, 2011), as in the martian *Meridiani Planum* (Klingelhöfer *et al.*, 2005).

**FIG. 1.**
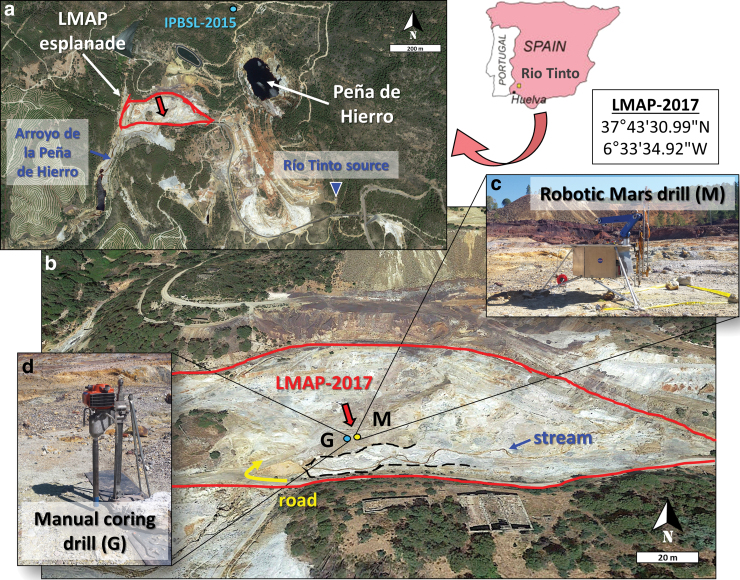
Site Drilling and study. Map of the study area in the Río Tinto basin (Southwestern Spain), with a regional map showing the location of the LMAP esplanade **(a)**, where the LMAP-2017 drilling and sampling campaign was conducted **(b)**. Close-up views of the Mars robotic drill **(c)** and manual coring drill **(d)** used for retrieving the Mars-drill cuttings (M, yellow dot) and coring drill samples (G, blue dot), respectively, are also shown. In **(a)**, it is indicated that a previous drilling project (IPBSL-2015) was conducted about 500 m northeast of the present sampling site (LMAP-2017). The black dashed shape in **(b)** highlights the white sulfate efflorescences arising along the stream. Satellite images are from Google Earth. LMAP, Life-detection Mars Analog Project. Color images are available online.

This study focuses on the northern domain of the Río Tinto basin, where the confluence of three acidic tributaries at *Peña de Hierro* (689 masl) constitute the source of the Río Tinto (Fernández-Remolar *et al.*, 2004). In particular, the present drilling simulation was conducted on an evaporitic esplanade formed around a little stream springing a few meters toward the southeastern from *Peña de Hierro* (Fig. 1a), where periodic flooding and drying of the terrain gives place to abundant sulfate efflorescences, such as those observed at the testing campaign time (Fig. 1b). Its easy access and the existence of geomicrobiological data on the area [*i.e*., from the MARTE, (Fernández-Remolar *et al.*, 2008; Stoker *et al.*, 2008) and IPBSL (Amils *et al.*, 2014) projects] were some of the reasons for selecting this site for the drilling simulation and bioanalytical survey.

Microbial life in Río Tinto is adapted to extreme conditions such as high acidity, abundance of heavy metals, and an Fe/S-based chemistry; acidophiles and chemolithotrophs are the major components of the microbial community. Eukaryotic microoorganisms contribute more than 60% of the Tinto basin biomass (López-Archilla *et al.*, 2001), where a significant proportion of them are photosynthetic (mainly chlorophytes), whereas the prokaryotic diversity is mainly contributed (ca. 80%) by three bacterial genera (*Leptospirillum* spp., *Acidithiobacillus ferroxidans*, and *Acidiphilium* spp.) members of the iron cycle (González-Toril *et al.*, 2003). As for the plants growing on the acidic soils of the Río Tinto basin (*e.g.*, *Pinus* sp., *Eucalyptus* sp., *Erica* sp., *Cistus ladanifer*, or *Taraxacum officinale*), most of them use different strategies to overcome physiological problems associated with the extreme conditions of the habitat, such as accumulation of metals in plant tissues.

### 2.2. Sample collection

In June 2017, the Trident 1m-class planetary prototype drill was tested on an acidic site in Río Tinto, rich in sulfur-iron material, with stickiness properties that may be similar to those on Mars. Automated robotic drilling was conducted on the LMAP evaporitic esplanade (Fig. 1), adjacent to an acidic stream carrying little water as expected for the summer season and flowing about 350 m away into a larger stream called *Arroyo de la Peña de Hierro* (Fig. 1). The lander-mounted system (automated drill, robotic arm, and full-scale Phoenix-like lander deck) was deployed at the Mars-analog site (37°43′30.99″N and 6°33′34.92″W) (Fig. 1c). The Trident drill developed by Honeybee Robotics Spacecraft Mechanisms Corporation (Brooklyn, New York) is a 15 kg, rotary-percussive and fully autonomous robotic drill rated for lunar temperatures, with its own AI software executive (Glass *et al.*, 2014). The drill power consumption was 30–40 W, 200 W max during 5–10-min drilling sequences. Drilling was conducted by using a bite-sampling approach, where samples were captured in 10–20 cm intervals to simulate a martian drilling scenario (Zacny *et al.*, 2013). All pieces involved in the drilling campaign were initially autoclaved in the laboratory and then sterilized once deployed in the field. Iterative drill and scoop cleaning with organic solvents (acetone, dichloromethane, and methanol) was performed before each new hole was drilled to avoid human-sourced or cross contamination.

**FIG. 2.**
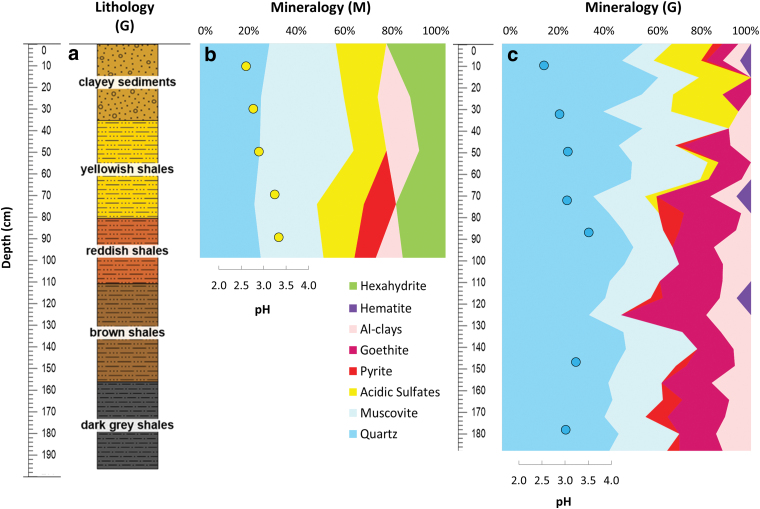
Stratigraphic column at the LMAP-2017 site, with the lithostratigraphic units reconstructed *de visu* on the manual coring drill (G) **(a)**, and the mineralogical composition of the materials retrieved from the Mars robotic drill (M) and manual coring drill (G) profiles **(b, c)**, based on X-ray diffraction analysis. In **(b**, **c)**, Al-clays comprise chlorite, vermiculite, montmorillonite, and kaolinite; whereas acidic sulfates include jarosite, natrojarosite, and copiapite. Al-clays, aluminous clays. Color images are available online.

The surface to drill was previously scraped with a solvent-cleaned (dichloromethane and methanol) metal brush and stainless-steel spatula. Subsurface samples (cuttings) were automatically acquired from the drilling profile (*i.e*., robotic *Mars drill* or “M”) at every 20-cm depth (surface—20, 20–40, 40–60, 60–80, and 80–100 cm) and autonomously transferred by the robotic arm to sterile polypropylene jars for offline analysis. In parallel, a second borehole profile (*i.e*., ground-truth or “G”) was drilled ∼2 m away from M down to 2-m depth with a CARDI EN 400, a manual drill (with a 3 kW gas-powdered motor, and 85-mm diameter commercial off-the-shelf diamond-impregnated coring drill bits and rods) for manual validation. G samples were taken from the manual coring drill at different depth intervals (*i.e*., 10–20, 35–40, 50–60, 75–80, 90–95, 150–155, and 180–188 cm), covering different stratigraphic layers or interphases according to the lithological reconstruction (Fig. 2a).

Physical splitting and subsample handling were conducted wearing nitrile gloves and using solvent-cleaned stainless-steel material (spatulas and scoops). A total of 12 discrete samples of robotic Mars drill cuttings (*n* = 5; 30–70 g each) and manual coring drill subsamples (*n* = 7 of 200–300 g each) were collected and stored cold (∼4°C) in the field and during their transport to the laboratory 7 days later. Once in the laboratory, all samples were frozen to −20°C and freeze-dried before geochemical and biochemical analysis. All freeze-dried M and G samples were powdered (mortar and pestle) and homogenized before taking aliquots for the different biogeochemical analyses.

### 2.3. Bulk geochemistry analysis

The mineralogical composition of the M drill and G drill samples was measured by using an Olympus Terra X-ray analyzer. The Terra is a field portable X-ray diffraction/X-ray fluorescence instrument based on technology developed for the CheMin instrument on the Mars Science Laboratory rover (Sarrazin *et al.*, 2005; Blake *et al.*, 2012). The bulk sample is ground with a mortar and pestle and filtered to less than 150 microns; then, 15 mg is placed in an acoustically vibrated chamber that presents the instrument optics with multifarious orientations of the crystalline structure. This results in an X-ray diffraction pattern that is free of the problematic preferred-orientation effects. The angular range is 5–50° 2θ with diffraction resolution of 0.25–0.30° 2θ FWHM.

Anions and low-molecular-weight organic acids were measured by ion chromatography (IC) in the water-extractable phase of the M and G samples. For this analysis, 2 g of sample was sonicated (3 × 1 min cycles), diluted in 10 mL of deionized water, and filtered through a 22 μm GF/F. The supernatants were collected and loaded into a Metrohm 861 Advanced compact ion chromatographer (Metrohm AG, Herisau, Switzerland) undiluted or at dilution values, depending on ion concentrations. For all the anions, the column Metrosep A supp 7–250 was used with 3.6 mM sodium carbonate (NaCO_3_) as eluent. The pH of the water solutions was measured with a pH meter (WTW, GmbH & Co. KG, Weilheim, Germany) after 24 h of solution stabilization. More details of the IC analysis are fully described in Sánchez-García *et al.* (2018).

Stable isotopic composition of the bulk organic carbon (δ^13^C) and total nitrogen (TN) (δ ^15^N) were measured on the robotic Mars drill and manual coring drill samples with isotope-ratio mass spectrometry (IRMS), following USGS methods (Révész *et al.*, 2012). Briefly, about 2 g of the subsurface samples was homogenized by grinding with a corundum mortar and pestle. To remove carbonates, HCl was added to the samples, let equilibrate for 24 h, and adjusted to neutral pH with ultrapure water. The residue was then dried (50°C) for 72 h; checked for a final, constant weight; and finally analyzed by IRMS (MAT 253; Thermo Fisher Scientific). The δ^13^C and δ ^15^N ratios were reported in the standard per mil notation by using three certified standards (USGS41, IAEA-600, and USGS40) with an analytical precision of 0.1‰. The content of total organic carbon (TOC % of dry weight [dw]) and TN (% dw) was measured with an elemental analyzer (HT Flash; Thermo Fisher Scientific) during the stable isotope measurements.

### 2.4. Lipid extraction, fractionation, and analysis

Lipids were extracted from the M and G samples with a method described in detail by Sánchez-García *et al.* (2019). Briefly, between 10 and 25 g of sample was extracted in a Soxhlet apparatus with a mixture of dichloromethane/methanol (DCM:MeOH, 3:1, v/v) during 24 h, after addition of three internal standards (tetracosane-D_50_, myristic acid-D_27_, 2-hexadecanol). After concentration to ∼2 mL and removal of elemental sulfur, the total lipid extracts (TLE) were separated into three fractions according to their different polarity (nonpolar, polar, and acidic). First, the TLE was passed through a Bond-Elut column (bond phase NH_2_, 500 mg, 40 μm particle size), where the neutral and acidic fractions were obtained by eluting with DCM:2-propanol (2:1, v/v) and subsequently acetic acid (2%) in diethyl ether, respectively. The neutral fraction was further separated into nonpolar and polar fractions by using an Al_2_O_3_ column (0.5 g of activated, 0.05–0.15-mm particle size Al_2_O_3_ in a Pasteur pipe), and eluting with hexane/DCM (9:1, v/v) and subsequently DCM/methanol (1:1, v/v), respectively. The acidic and polar fractions were trans-esterified (BF_3_ in methanol) and tri-methylsilylated (N,O-bis [trimethylsilyl] trifuoroacetamide [BSTFA]), respectively, before their analysis as fatty acid methyl esters (FAMEs) and tri-methyl silyl alkanols by gas chromatography–mass spectrometry (GC-MS).

The three lipid fractions were analyzed by GC-MS using a 6850 GC system coupled to a 5975 VL MSD with a triple axis detector (Agilent Technologies) operating with electron ionization at 70 eV and scanning from *m/z* 50 to 650. The analytes were injected (1 μL) and separated on a HP-5MS column (30 m × 0.25 mm i.d. × 0.25 μm film thickness) by using He as a carrier gas at 1.1 mL min^−1^. The oven temperature was programmed from 50°C to 130°C at 20°C min^−1^ and then to 300°C at 6°C min^−1^ (held 20 min) for the nonpolar fraction (*i.e*., hydrocarbons). For the polar (*i.e*., alkanols and sterols) and acidic (*i.e*., fatty acids) fractions, the oven temperature was programmed from 70°C to 130°C at 20°C min^−1^ and to 300°C at 10°C min^−1^ (held 10 min for the acidic or 15 min for the polar fractions). The injector temperature was 290°C, the transfer line was 300°C, and the MS source was 240°C.

Compound identification was conducted by comparing mass spectra and/or reference materials on mass-to-charge ratios of 57 (*n*-alkanes and isoprenoids), *m/z* = 74 (*n*-fatty acids as FAMEs), and *m/z* = 75 (*n*-alkanols and sterols). For compound quantification we employed external calibration curves of *n*-alkanes (C_10_–C_40_), *n*-FAME (*i.e.,* FAMEs, C_8_–C_24_), *n*-alkanols (C_10_, C_14_, C_18_, and C_20_), and branched isoprenoids (2,6,10-trimethyl-docosane, crocetane, pristane, phytane, squalane, and squalene), all from Sigma Aldrich. The recoveries of the internal standards were measured to average 72% ± 23%.

### 2.5. DNA extraction, polymerase chain reaction amplification, and DNA sequencing

Total genomic DNA was extracted from the M and G samples by using the commercial kit PowerMax Soil DNA Isolation Kit (MO BIO Laboratories, Inc.), following the manufacturer's instructions with a 1-h (room temperature) enlargement of the lysis step. Polymerase chain reaction (PCR) amplification was then carried out on bacterial 16S rDNA V3–V4 gene (using the primer pairs 341-F/805-R, Herlemann *et al.*, 2011) following Warren-Rhodes *et al.* (2018) specifications. Illumina MiSeq sequencing (Illumina, Inc., San Diego, CA) was then employed to construct a paired-end amplicon library.

Amplicon reads were processed with MOTHUR software v.1.40.0 (Schloss *et al.*, 2009), by using a custom script based on MiSeq SOP (Kozich *et al.*, 2013). Briefly, the pipeline consisted of several reads-trimming steps based on both quality and length (>400 bp) criteria. After trimming, sequence reads were clustered into operational taxonomic units (OTUs) at 97% of sequence similarity level. Even sequencing depth—corresponding to the lesser number of sequences found within the samples, that is, 63,736—was obtained by independent rarifying of datasets by random selection.

OTU representative sequences were then compared against the RDP database (RDP reference files v.16; release 11) (Cole *et al.*, 2014) for taxonomic affinities assignment. Those OTU sequences reported as “cyanobacteria/chloroplast” were further assigned a taxonomic identity by comparing them against nr/nt (NBCI), EMBL, Greengenes, and SILVA databases for more precise cyanobacteria taxonomic identification. The sequences assigned to “mitochondria” or “chloroplast” were removed from further analyses. Community parameters, such as phylogenetic richness (*S*, total number of OTUs) and Shannon diversity index (*H*′), were calculated on the samples by using R package “vegan” (Oksanen *et al.*, 2017). The same statistical package was also employed to perform a correspondence analysis (CA) for microbial classes and bulk geochemistry parameters (standardized and log-normalized).

### 2.6. Multiplex fluorescent sandwich microarray immunoassay

Powdered subsurface M and G samples were analyzed by a fluorescent sandwich microarray immunoassay (FSMI) with the LDChip immunosensor (*i.e*., LDChip) (Rivas *et al.*, 2008; Parro *et al.*, 2008; 2011). The LDChip is a shotgun antibody microarray produced for the simultaneous detection of potential microbial biomarkers from environmental samples (Parro *et al.*, 2018; Sanchez-García *et al.*, 2019) and/or for detecting possible traces of life in the field of the planetary exploration (Blanco *et al.*, 2017; Moreno-Paz *et al.*, 2018) as part of the SOLID instrument concept (Parro *et al.*, 2008, 2011).

The LDChip is composed of 200 polyclonal antibodies produced against a wide range of immunogens: small organic molecules, peptides and proteins, and other biopolymers (exopolysaccharides, lipopolysaccharides), spores, or whole cells (bacteria and archaea) from extant or well-preserved remains of extinct life (Rivas *et al.*, 2008; Sanchez-García *et al.*, 2018). The antibodies used in this work are described in detail elsewhere (Sanchez-García *et al.*, 2018, and in table S1 supplementary materials in Sánchez-García *et al.*, 2019). In addition, bovine serum albumin, protein printing buffer, and preimmune sera were used as negative controls. To build the microarray, the immunoglobulin (IgG) fraction of each antibody was purified with protein-A by using the PURE1A kit (Sigma Aldrich Quimica S.L.) and printed onto microscope slides in a triplicate spot-pattern. Antibodies were titrated, fluorescently labeled with Alexa 647, and used to perform the FSMI as reported in Rivas *et al.* (2008).

In this study, Río Tinto subsurface samples were analyzed in the field with the LDChip immunosensor as previously described (Rivas *et al.*, 2008; Parro *et al.*, 2011; Blanco *et al.*, 2012). Briefly, 0.5 g of each sample was suspended in 2 mL of TBSTRR buffer (0.4 M Tris-HCl pH 8, 0.3 M NaCl, 0.1% Tween 20), homogenized with a handheld ultrasonicator to extract the organic matter, and finally filtered through a 5-μm filter. A volume of 50 μL of each filtrate was used for the FSMI as a multianalyte-containing sample in the multiarray analysis cassette as previously described (Rivas *et al.*, 2008; Blanco *et al.*, 2017; Moreno-Paz *et al.*, 2018). After a washing step, 50 μL of the mixture of 200 alexa-647 labeled antibodies was incubated in each chamber and then scanned in the GenePix 4100A scanner and the resulting images were analyzed and quantified by the GenePix Pro 7.0 Software (Molecular Devices, Sunnyvale, CA). The fluorescence intensity (F) of positive antigen-antibody reactions was calculated as reported by Rivas *et al.* (2011). To avoid false positives in the FSMI, fluorescent signal intensities for each antibody were considered positives when they had intensity at least 2.5 times the background level.

## 3. Results

### 3.1. Profiling the geochemistry of 1-m depth robotic drill in Río Tinto sediments

A simulated Mars drill (M) down to 1 m was performed with a rotary-percussive and fully autonomous robotic drill prototype on iron/sulfur-rich sediments in an evaporitic material about 10 m apart from the river stream (Fig. 1). As a ground-truth experiment, a second drill (G) was carried out ∼2 m far from M, by using a commercial, manual drilling and coring system (Section 2). The M drill rendered samples with a few tens of grams (20–70 g) of material, whereas G allowed us to collect hundreds of grams (Table 1) so that the mass required for multiple analyses at the different depths was not a limitation.

**Table 1.** Concentration of Inorganic and Organic Anions (μg·g^−^^1^) in the Subsurface Samples Along the Robotic Mars (M) and Manual Coring (G) Drills

Depth intervals (cm)	M drill					G drill						
	0–20	20–40	40–60	60–80	80–100	10–20	35–40	50–60	75–80	90–95	150–155	180–188
Sample size (g)	27	47	49	45	53	163	142	159	153	207	170	158
Sulfate	78	72	71	68	56	40	43	41	35	35	48	44
Chloride	0.36	0.18	0.18	0.19	0.17	0.16	0.14	0.17	0.14	0.18	0.15	0.17
Fluoride	0.10	0.10	0.10	0.11	0.10	0.045	0.094	0.093	0.091	0.10	0.11	0.10
Bromide	0.079	n.d.	n.d.	n.d.	n.d.	n.d.	n.d.	n.d.	n.d.	n.d.	n.d.	n.d.
Nitrate	n.d.	n.d.	0.011	n.d.	n.d.	n.d.	n.d.	n.d.	n.d.	n.d.	n.d.	n.d.
Phosphate	n.d.	n.d.	n.d.	n.d.	n.d.	n.d.	n.d.	n.d.	0.22	n.d.	n.d.	n.d.
Acetate	0.022	0.023	0.036	0.041	0.026	n.d.	0.047	n.d.	n.d.	n.d.	0.027	0.024
Formate	0.031	0.033	0.040	0.044	0.043	n.d.	0.085	0.056	0.049	0.048	0.039	0.034

n.d. = not detected.

The X-ray diffraction analysis revealed a subsurface mineralogy primarily composed of quartz and muscovite (Fig. 2). The relative abundance of both mineral species varied between the robotic M drill and manual coring G drill profiles, with muscovite being dominant (25–38%) along M (Fig. 2b) and quartz (35–63%) along G (Fig. 2c). Acidic sulfate minerals, including jarosite, natrojarosite, and copiapite, were similarly abundant throughout the M drill (13–21%) and in the upper layers (down to 80 cm) of the G profile (3–27%). Both M and G set of samples contained a ubiquitous comparable proportion (11–13% versus 4–18%, respectively) of aluminous clays (*i.e*., chlorite, vermiculite, montmorillonite, and kaolinite). In contrast, pyrite was only patchy throughout the two profiles and hematite was only detected in the G drill. Hexahydrite and goethite were distributed ubiquitously throughout the M (11–24%) and G (6–34%) profiles, respectively (Fig. 2).

**FIG. 3.**
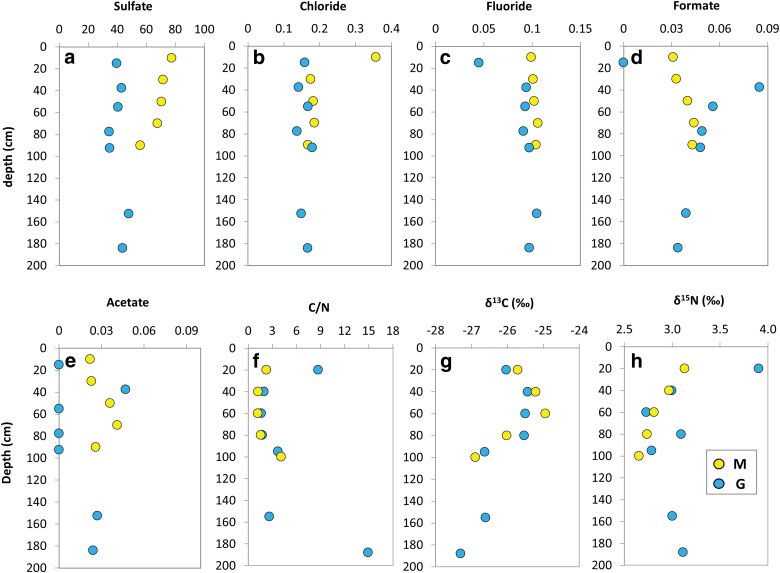
Depth distribution of bulk (elemental, molecular, and isotopic) geochemical variables in the Mars robotic drill (M, yellow) and manual coring drill (G, blue) profiles. Scatterplots showing the concentration (μg·g^−1^) of inorganic **(a–c)** and low mass-organic anions **(d, e)**; atomic C/N ratio **(f**; dimensionless**)**, as well as organic carbon **(g)** and total nitrogen **(h)** isotopic ratios (per mil). Color images are available online.

The IC analysis detected the presence of diverse soluble inorganic and organic anions (Table 1). Sulfate was the most abundant anion among both the M (56–78 μg·g^−1^) and G (35–48 μg·g^−1^) samples. Chloride and fluoride were similarly abundant throughout the two drills (Fig. 3b, c) whereas bromide, nitrate, and phosphate were only present at low concentrations (≤0.215 μg·g^−1^) in a few depths of either the M or G profiles (Table 1). Formate showed concentrations ranging from 0.031 to 0.044 μg·g^−1^ in the M samples, and from 0 to 0.085 μg·g^−1^ in the G series (Fig. 3d). Acetate was detected to be ubiquitous throughout the M profile (0.022–0.041 μg·g^−1^), but only patchy (0–0.047 μg·g^−1^) along G (Fig. 3e).

The TOC content ranged from 0.12% to 0.38% (wt/wt) of dw among M and from 0.12% to 1.3% (wt/wt) among G (Table 2). The TN content ranged from 0.067% to 0.10% (wt/wt) in G and from 0.078% to 0.10% (wt/wt) in M, resulting in generally similar ratios of TOC to TN ratios (*i.e*., C/N) in both the M and G profiles (Fig. 3f). The carbon isotopic ratio (δ^13^C) varied from −27.3‰ to −25.4‰ in G and from −26.9‰ to −24.9‰ in M, whereas the nitrogen isotopic ratio from 2.7‰ to 3.9‰ in G and from 2.7‰ to 3.1‰ in M (Fig. 3g, h).

**Table 2.** Concentration (ng·g^−^^1^ of Dry Weight) of Lipid Biomarkers and Bulk Elemental and Stable Isotopic Composition of Organic Matter in the Subsurface Samples Along the Robotic Mars (M) and Manual Coring (G) Drills

		M drill					G drill						
m/z	Depth intervals (cm)	0–20	20–40	40–60	60–80	80–100	10–20	35–40	50–60	75–80	90–95	150–155	180–188
	TOC (% dw)	0.18	0.13	0.12	0.15	0.38	0.74	0.20	0.15	0.12	0.32	0.18	1.13
	TN (% dw)	0.08	0.10	0.10	0.10	0.09	0.09	0.10	0.09	0.07	0.09	0.07	0.08
	C/N^a^	2.3	1.2	1.2	1.6	4.1	8.7	2.0	1.6	1.8	3.7	2.6	14.9
	δ^13^C (‰)	−25.7	−25.2	−24.9	−26.0	−26.9	−26.0	−25.4	−25.5	−25.5	−26.6	−26.6	−27.3
	δ^15^N (‰)	3.13	2.97	2.81	2.74	2.65	3.90	2.99	2.73	3.09	2.79	3.00	3.11
57	*n*-alkanes	1132	311	538	156	144	1006	3204	669	721	1504	9164	526
	ACL_*n*-alkanes_ (C_10–_C_40_)^b^	29	28	25	28	28	29	32	28	24	22	31	25
	CPI (C_21–_C_31_)^c^	1.1	0.87	0.029	0.35	0.35	1.7	0.64	0.42	3.8	0.00	0.026	0.27
	oddHMW/evenLMW^d^	4.3	2.8	n.d.	1.3	0.74	5.7	3.3	0.77	n.d.	0.0031	0.022	n.d.
	Pristane	1.0	n.d.	0.58	1.6	0.00	1.1	0.57	1.3	n.d.	n.d.	n.d.	4.3
	Phytane	n.d.	n.d.	n.d.	n.d.	n.d.	n.d.	0.28	n.d.	0.04	n.d.	9.1	7.9
	Squalane	17	6.0	2.9	8.3	4.2	n.d.	0.00	1.8	0.00	n.d.	3.0	4.5
74	*n*-fatty acids	904	492	215	892	880	858	586	400	1320	966	156	129
	ACL_*n*-fatty acids_ (C_6_–C_33_)^b^	26	22	26	22	22	24	23	22	21	19	14	21
	*i-/a-*fatty acids (C_11_–C_29_)^e^	33	27	11	59	62.1	27	18	15	223	8.8	45	42
	C_16:1_ (ω7)^f^	n.d.	n.d.	n.d.	n.d.	n.d.	0.41	0.28	0.00	1.2	0.98	1.6	0.13
	C_18:1_ (ω7/9)^g^	n.d.	n.d.	1.4	4.5	5.1	0.24	0.034	0.0060	n.d.	n.d.	7.8	0.17
	C_18:2_ (ω6, ω9)^h^	n.d.	0.33	n.d.	n.d.	n.d.	0.53	0.61	0.43	24	23	n.d.	n.d.
75	*n*-alkanols	4702	2844	468	21,363	253	901	2715	866	1178	209	2371	150
	ACL_*n*-alkanols_ (C_10_–C_30_)^b^	26	25	26	32	25	24	24	23	24	17	21	25
	Campesterol^i^	n.d.	n.d.	0.3	n.d.	0.2	5.9	n.d.	n.d.	9.8	n.d.	n.d.	n.d.
	Stigmasterol^j^	n.d.	n.d.	n.d.	n.d.	n.d.	2.8	n.d.	n.d.	n.d.	n.d.	n.d.	n.d.
	β-sitosterol^k^	29	15	4.7	203	1.8	21	n.d.	1.5	24	18	n.d.	2.7

^a^ Ratio of TOC over TN.

^b^ ACL of C_10_–C_40_ *n*-alkanes; C_6_–C_33_ *n*-fatty acids; and C_10_–C_30_ n-alkanols. *ACL*_i-*n*__=_ ∑(i·X_i_ +…+ n·X_n_)/∑X_i_ +…+ X_n_), where X is concentration (van Dongen *et al.*, 2008).

^c^ CPI, CPI _i-*n*__=_ ½ Σ(X_i_+X_i+2_+…+X_n_)/Σ(X_i−1_+X_i+1_+…+X_n−1_) + ½Σ(X_i_+X_i+2_+…+X_n_)/Σ(X_i+1_+X_i+3_+…+X_n+1_), where X is concentration (van Dongen *et al.*, 2008).

^d^ Ratio of odd, HMW alkanes (C_27_, C_29_, and C_31_) over even, LMW alkanes (C_16_, C_18_, and C_20_).

^e^ Sum of *iso-* and *anteiso- n*-fatty acids (C_11_–C_29_).

^f^ 9-Hexadecenoic acid (measured as hexadec-9-enoate that is a fatty acid methyl ester).

^g^ 9/11-Octadecenoic acid (measured as octadec-9/11-enoate).

^h^ 9,12-Octadecenoic acid (measured as octadec-9,12-enoate).

^i^ 24-Methylcholest-5-en-3β-ol.

^j^ 24-Ethylcholest-5,22-dien-3β-ol.

^k^ 24-Ethylcholest-5-en-3β-ol.

ACL = average chain length; CPI = carbon preference index; dw = dry weight; HMW = high molecular weight; LMW = low molecular weight; TN = total nitrogen; TOC = total organic carbon.

### 3.2. Lipid biomarker profiles

**FIG. 4.**
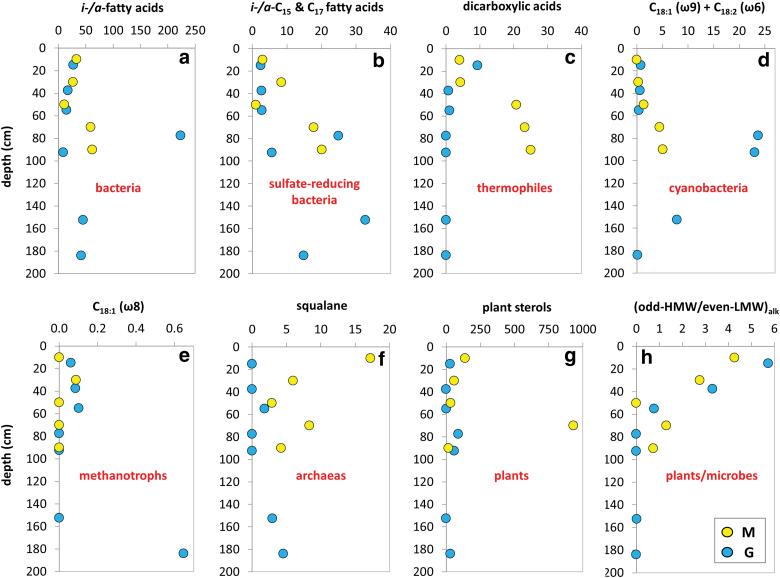
Depth distribution of lipid biomarkers along the Mars robotic drill (M, yellow) and manual coring drill (G, blue) profiles. Concentration (ng·g^−1^dw) of lipids, biomarkers of bacteria as *iso*-/*anteiso*-fatty acids of C_11_–C_29_ **(a)**; sulfate-reducing bacteria as sum of *iso*-/*anteiso*-C_15_ and C_17_ fatty acids **(b)**; thermophiles as sum of dicarboxylic acids C_6_–C_30_ **(c)**; cyanobacteria as sum of C_18:1_ (ω9) and C_18:2_ (ω6,9) fatty acids **(d)**; methanotrophs, as C_18:1_ (ω9) fatty acids **(e)**; archaea as squalane **(f)**; higher plant sterols as sum of campesterol, stigmasterol, and β-sitosterol **(g)**; and proxy of relative abundance of plant versus microbial biomass as ratio of odd, high molecular weight (C_27_, C_29_, and C_31_) over even, low molecular weight (C_16_, C_18_, and C_20_) alkanes **(h)**. Note that the source assignation should be taken with caution assuming that the inference corresponds to the most likely but not exclusively attributed sources to the detected biomarkers according to the literature. dw, dry weight. Color images are available online.

The GC-MS analysis of TLE from M and G set of samples yielded molecular biomarkers within the families of *n*-alkanes, isoprenoids, FAMEs, *n*-alkanols, and sterols. Straight chain fatty acids (a.k.a. *normal* or *n*-fatty acids) of 6 to 33 carbons were measured at concentrations from 129 to 1320 ng·g^−1^ of dw (Table 2). They showed average chain length (ACL) ranging from 14 to 26. *n*-Fatty acids with one [*e.g*., C_16:1_ (ω7) or C_18:1_ (ω7/9)] or two [C_18:2_ (ω6,9)] unsaturations were also detected (Table 2). Branched fatty acids with *iso-* and *anteiso* configuration (*i-/a-*) were detected between C_11_ and C_29_ at concentrations from 9 to 223 ng·g^−1^ dw (Fig. 4a), with predominance of the *i*/*a*-C_15_ and *i*/*a*-C_17_ compounds (Fig. 4b). Fatty acids with two carboxyl groups (*i.e*., dioic or dicarboxylic acids) from 6 to 30 carbons were also found at concentrations ranging from 0.7 to 173 ng·g^−1^ dw (Table 1 and Fig. 4c).

Straight chain alkanols (*i.e*., *n*-alkanols) of ACL ranging from 17 to 32 were measured in the *m/z* = 75 ion at concentrations from 150 to 21,363 ng·g^−1^ dw, together with terrestrial sterols (Goad and Akihisa, 1997) such as campesterol, stigmasterol, and β-sitosterol (3.6–1194 ng·g^−1^ dw; Table 2 and Fig. 4g).

Straight chain alkanes (*n*-alkanes) with between 10 and 40 carbons were measured at concentrations from 144 to 9164 ng·g^−1^ dw (Table 2). They exhibited molecular distribution with dominance of the high-molecular-weight (HMW) homologs that produced ACL between 22 and 32 (Table 2). The carbon preference index of the *n*-alkanes was generally lower than one in the two profiles, except at the 0–20 cm (M and G) and 75–80 cm (G) depth intervals. The ratios of vegetal, odd HMW (*i.e*., C_27_, C_29_, and C_31_) over microbial, low-molecular-weight (LMW) *n*-alkanes (Eglinton and Hamilton, 1967), both odd LMW (C_17_, C_19_, and C_21_) and even LMW (C_16_, C_18_, and C_20_), were generally larger than the ones throughout both drills down to the 80–100-cm interval (Fig. 4h). Isoprenoids such as pristane, phytane, and squalane were detected in the same ion as the *n*-alkanes (*i.e*., *m/z* = 74) at total concentrations ranging from 0.037 to 18 ng·g^−1^ dw (Table 2).

### 3.3. Prokaryotic diversity by DNA sequencing

**FIG. 5.**
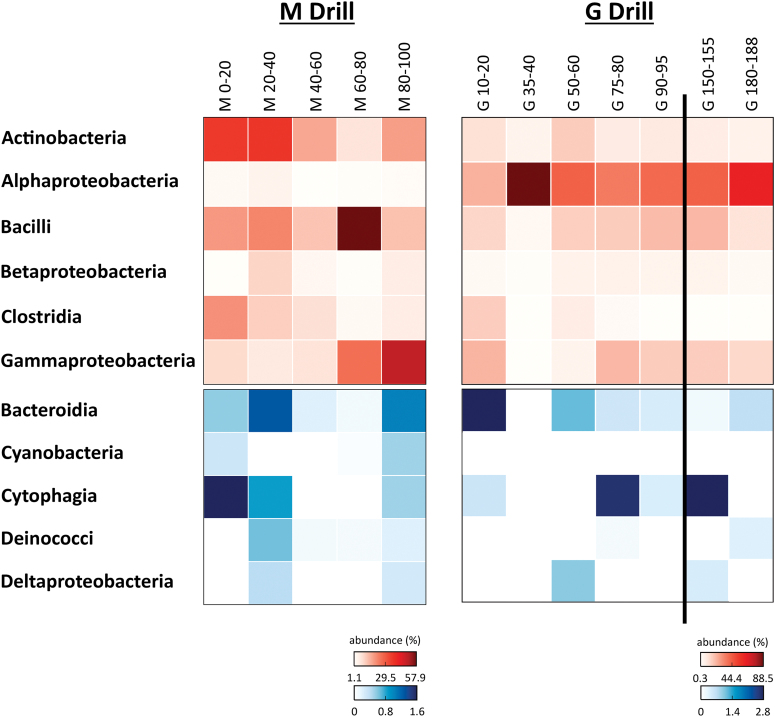
Bacterial DNA diversity. Heat maps showing the relative abundance (number of sequences at the class level) along the Mars robotic drill (M) and manual coring drill (G) profiles. Only bacterial classes accounting for >0.5% of total number of sequences are plotted. Both heat maps are split into two to better show the relative abundance of the less represented classes (blue scale). In both the red and blue scales, white color indicates absence of sequences (please notice the different % of abundance in each heat map). Color images are available online.

Total DNA was extracted from both set of samples, used for PCR amplification of the 16S rRNA gene, sequencing, and bioinformatic analysis. The results indicated that the bacterial OTUs adscriptions along the drills were largely represented by Proteobacteria, Firmicutes, and Actinobacteria phyla, together accounting for 85% and 93% of the total bacterial reads in the M and G drills, respectively. Along the M drill, Actinobacteria and Clostridia classes were more abundant (21% of bacterial reads in these samples) in the upper layers, whereas Bacilli and Gammaproteobacteria were more abundant with depth (21%) (Fig. 5a). Other classes such as Alpha- and Betaproteobacteria represented only 1.9% and 3.3% of the sequences found at the M drill. The microbial community in the G drill was rather dominated by Alphaproteobacteria (48%), with minor presence of the other classes mentioned earlier (Fig. 5b). In both the M and G drills, Cytophagia, Bacteroidia, Deltaproteobacteria, Deinococci, and Cyanobacteria classes were found at minor proportion (<1%) (Fig. 5).

**FIG. 6.**
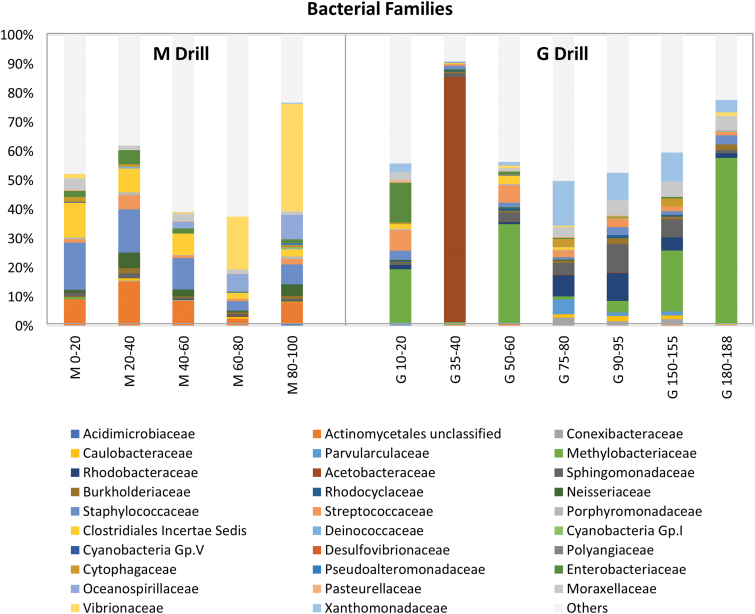
Comparison of bacterial community composition along the robotic Mars drill (M) versus manual coring drill (G) profiles. Relative abundance (number of sequences) of bacterial families along the M and G profiles. Only the most abundant family within each order is represented. Less abundant families are grouped in the “others” category (light gray bars). Color images are available online.

Within the described classes, the most abundant family along the M drill was Staphylococcaceae (Bacillales order, Bacilli class; 3–16% of bacterial reads). This family showed greater abundance in the upper sections of M, along with unclassified families belonging to the orders Actinomycetales (Actinobacteria; 2–15%) and Clostridiales (Firmicutes; 2–12%) (Fig. 6). In contrast, the families Oceanospirillaceae (Oceanospirillales, Gammaproteobacteria; 0.003–8%) and Vibrionaceae (Vibrionales, Gammaproteobacteria; 0.02–37%) were relatively more abundant at deeper layers. Neisseriaceae (Neisseriales, Betaproteobacteria; 0.6–5% of bacterial reads) and Moraxallaceae (Pseudomonadales, Gammaproteobacteria; 1–4%) were families less abundantly represented in M (Fig. 6).

The bacterial community at the G drill showed a slightly different structure, with a widespread distribution of Methylobacteriaceae (Rhizobiales, Alphaprotebacteria; 0.9–57%) that showed its highest abundance at the deepest section (Fig. 6). A comparable widespread distribution, but accounting for a much lower number of bacterial reads, was that of Xanthomonadaceae (Xanthomonadales, Gammaproteobacteria; 0–15%), Sphingomonadaceae (Sphingomonadales, Alphaproteobacteria; 0.9–10%), Rhodobacteraceae (Rhodobacterales, Alphaproteobacteria; 0.002–10%), Moraxallaceae (Pseudomonadales, Gammaproteobacteria; 0.002–5%), and Streptococcaceae (Lactobacillales, Bacilli; 0.6–7%). From those, Streptococcaeae was more abundant in the upper layers of the G drill, whereas Xanthomonadaceae, Sphingomonadaceae, Rhodobacteraceae, and Moraxallaceae were preferentially distributed at depth (Fig. 6). Of special interest was the particularly high abundance (84% of sequences) of the family Acetobacteraceae (Rhodospirillales, Alphaproteobacteria) in almost exclusively the 35–40-cm depth interval in G (Fig. 6).

**FIG. 7.**
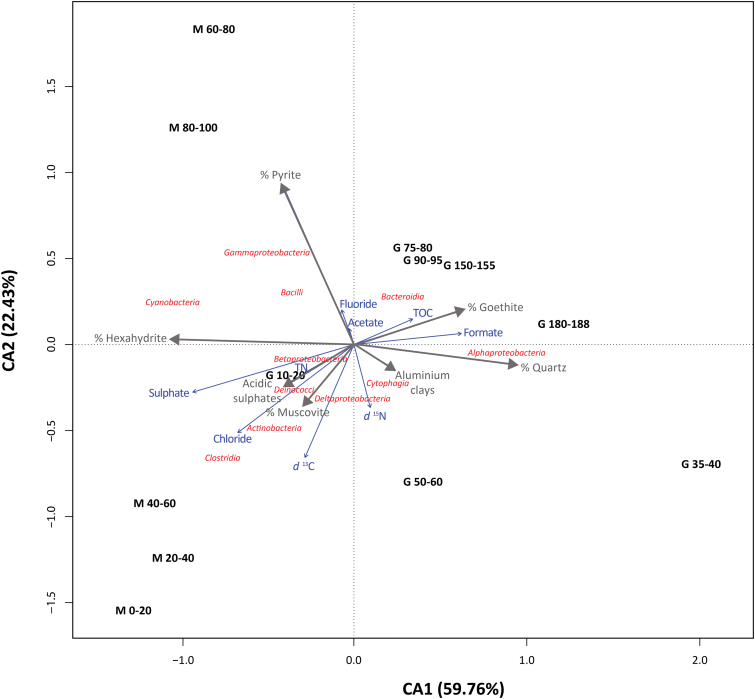
Single CA of bacterial classes (red) from different sampling depths (black) along the robotic drill (M) and manual coring drill (G) profiles with mineralogical (gray) and geochemical variables (blue). Only bacterial classes accounting for >0.5% of total number of sequences are plotted. In total, CA1 and CA2 explained 82.19% of the dataset's variance. The closer the clustering of microbial groups and/or mineralogical/geochemical variables to a sampling profile and depth, the more characteristic these groups are at that site and the more explained by the local mineralogy/geochemistry they are. Distance among sampling sites depicts compositional differences between them, and arrow length of the participation of each geochemical/mineralogical variable in explaining the variance. CA, correspondence analysis. Color images are available online.

The CA of bacterial classes and mineralogy-geochemistry (Fig. 7) showed that all samples belonging to the same drill clustered together along the CA1 axis (explaining 59.8% of variance). Only the most surficial sample from the G drill (10–20-cm depth) was positioned near the M samples. CA2 accounted for 22.4% of total variance explained by the analysis, and it grouped the samples according to a depth pattern (Fig. 7). Most of the bacterial classes were located close to the ordination center, with Alphaproteobacteria clearly clustering along with the G samples, Clostridia and Actinobacteria located close to M samples from the upper layers, and Gammaproteobacteria and Bacilli located close to deep M layers. Samples from the M profile showed higher influence from geochemical features such as δ^13^C, chloride, or pyrite, muscovite and acidic sulfates; whereas those from G were rather affected by the amount of quartz, goethite, TOC, or aluminous clays.

**FIG. 8.**
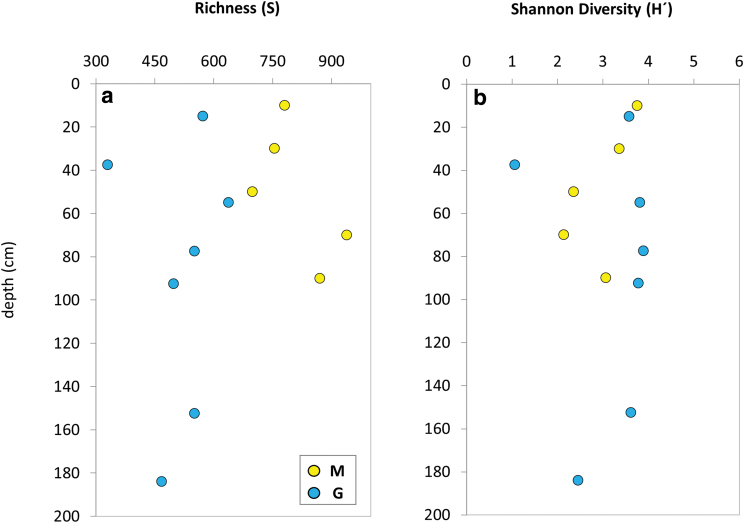
Estimation of microbial richness **(a)** and diversity **(b)** at different depths along the robotic Mars drill (M, yellow dots) and manual coring drill (G, blue dots) profiles. Richness **(a)** was based on the total number of OTUs (*S*) and diversity **(b)** on the Shannon index (*H*), calculated following the formula employed in R package “vegan” (Oksanen *et al.* 2017) defined as $H^{\prime }=-\sum_{i=1}^{S}p_{i}log_{b}p_{i}$, where *S* is the species richness, $p_{i}$ is the proportional abundance of species *i*, and *b* is the base of the natural logarithm. Color images are available online.

Samples from the M drill contained a higher number of OTUs (*i.e*., *S* ranging from 699 to 939) than those from the G drill (*S* = 330–638). This resulted in the Shannon diversity index (*H*′) ranging from 2.14 to 3.76 in M and from 1.07 to 3.90 in G. In both drills, the bacterial community richness (*S*) decreased from the surface to 20–40 cm depth in G, or 40–60 cm in M, showing a maximum at 40–60 cm in G or 60–80 cm in M (Fig. 8a). The microbial diversity (*H*′) displayed a general decrease along the M drill, with an upturn in the deepest interval (Fig. 8b). Overall, the *H*′ index varied little throughout the G drill, except for the 35–40- and 180–188-cm depths showing lower values.

### 3.4. Microbial markers profiling with an LDChip immunoassay

**FIG. 9.**
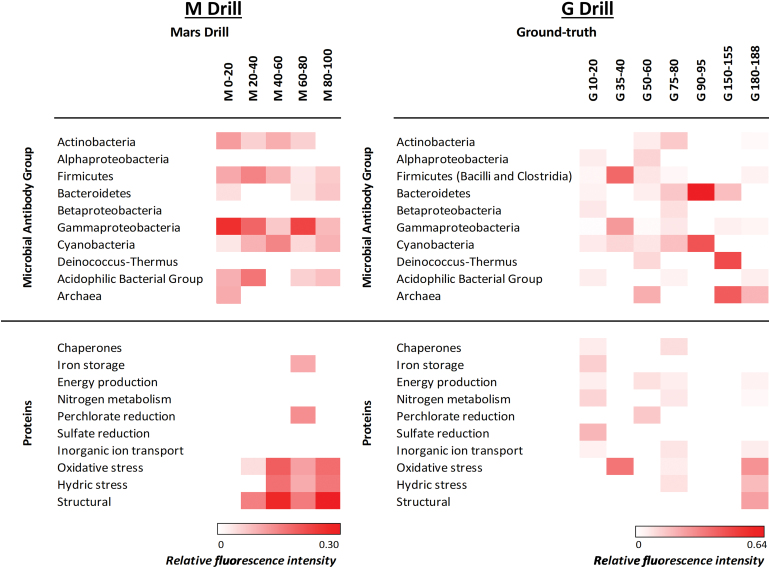
Heat map representing relative fluorescence signals obtained in the M and G samples with fluorescent sandwich microarray immunoassay in the LDCHip microarray immunosensor. Antibodies were grouped in different categories corresponding to main prokaryotic groups and proteins involved in several potential metabolisms. Antibodies printed in the microarray are described in Section 2. Proteins detected with the LDChip in this work are as follows: (1) chaperones: HscA and GroEL; (2) iron storage protein BFR (bacterioferritin); (3) energy production: Bacteriorodopsin, RRO (rubredoxin), NADH Deshidrogenase quinone, and OmcS (C-type cytochrome); (4) nitrogen metabolism: NifD, NifS, NirS (nitrite reductase), and NRA (nitrate reductase); (5) perchlorate reduction: perchlorate reductase; (6) sulfate reduction: DsrA (dissimilatory sulfite reductase subunit A); (7) inorganic ion transport: ModA and K+ tranporter; (8) oxidative stress: CcdA (cytochrome c-type biogenesis), PhcA2 (phycocyanin alpha-subunit), DhnA2 (fructose-bisphosphate aldolase), and PufM2 (photosynthetic reaction center M subunit); (9) hydric stress: SodA and SodF (superoxide dismutases) and WshR (water stress protein); and (10) structural: FtsZ (cell division protein). Relative fluorescence intensity of each category was calculated as the average of the fluorescence intensity for each antibody in three replicate samples and considering no redundant positive immunodetections for each category. Relative fluorescence intensity was plotted in a color gradient scale ranging from 0 to 1 (white represented values below the limit of detection and red values for the maximum fluorescence intensity obtained for each drill profile). LDChip, life detector chip. Color images are available online.

Up to 0.5 g of each sample from the two drills was processed and analyzed for detecting microbial markers by FSMIs (Section 2). The fluorescence intensity for each antibody spot in the LDChip microarray was quantified, corrected by the background and blank control, and represented as a heat map showing the relative fluorescence intensity of the positive immunodetections by the LDChip (Fig. 9).

In the M drill, the LDChip showed positive immunodetections of Gammaproteobacteria (mainly *Acidithiobacillus* spp, *Psicrobacter* sp., and *Halothiobacillus* sp.), Firmicutes (mainly *Bacillus subtilis* spores), Actinobacteria (both mycelium and spores of *Streptomyces*), and Cyanobacteria (*Anabaena* spp.). Also, positive signals from Bacteroidetes and cellular and exopolysaccharide extracts from Río Tinto (streams and biofilms; González-Toríl *et al.*, 2003), here named as “acidophilic bacterial group,” were also observed at certain depths (Fig. 9a). This group was dominated by acidophilic iron oxidizers such as *Leptospirillum* spp. (Nitrospirae), *Acidithiobacillus* spp. (Gammaproteobacteria), and *Acidimicrobium* spp. (Actinobacteria). In addition, the sulfide-oxidizing bacterium *Sulfobacillus acidophilus* (Firmicutes) was also detected throughout the M drill. Archaeal markers (*Halorubrum* spp., and *Pyroccoccus* spp.) were detected only in the most-shallow sample of the M drill.

In the G drill, the LDChip showed a patchy distribution with no particular pattern with depth, except Bacteroidetes, Cyanobacteria (*Anabaena* sp. and *Microcystis* sp.), *Deinococcus-Thermus*, and Archaea (*Halorubrum* spp., and *Pyroccoccus* spp.) that were slightly more abundant in deeper samples (Fig. 9b). The “acidophilic bacterial group” was only detected in some G samples always at a lower fluorescence signal intensity than in the M drill (Fig. 9). Finally, iron reducers such as *Acidocella* sp. and *Acidiphilium* sp. (Alphaproteobacteria), the anaerobic sulfate-reducing bacterium *Desulfosporinus meridiei* (Firmicutes), and the chlorate-reducer *Ideonella* spp. (Betaproteobacteria) were also identified by the LDChip overall in the upper layers of the G drill.

The LDChip also showed positive immunodetection of proteins involved in relevant metabolic processes such as hydric stress (water stress-resistance protein WshR or superoxide dismutase proteins SodA and sodF) or oxidative stress (antioxidant protein PhcA2, which is phycocianin α-chain from *Nostoc*), mostly in the M drill (Fig. 9a). Other proteins involved in septation and sporulation processes in *Streptomyces* and *Bacillus* such as the FtsZ (Beall and Lutkenhaus, 1991) or CcdA (Schiott and Hederstedt, 2000) were detected in M from 20-cm depth. In the G drill, the proteins related to oxidative and hydric stress (SodA, sodF, and WhsR) were only detected at 75–80- and mostly 180–188-cm-depth intervals (Fig. 9b). Other proteins detected and irregularly distributed in G were chaperone-like proteins (*i.e*., HscA and GroEL) or those involved in energy metabolism (*i.e*., NADH dehydrogenase quinone, bacteriorhodopsin, rubredoxin, and cytochromes), nitrogen fixation (*i.e*., NifD and NifS of *Leptospirillum* sp.), nitrate reduction (*i.e*., nitrate reductase, or NRA of *Micrococcus* spp.), and nitrite reduction (nitrite reductase or NirS protein) (Fig. 9b).

## 4. Discussion

### 4.1. Multianalytical biomarker profiling along a Mars simulated drill

The analysis of different molecular biomarkers revealed a ubiquitous presence of microbial biomass throughout the 1m-depth M drill profile. On one hand, there was a generalized detection of *i-/a-* fatty acids (Fig. 4a) typically associated to bacterial sources (Kaneda, 1991), consistent with low C/N ratios (Fig. 3f). Organic matter with C/N ratios between 4 and 10 (here ≤4.1) reflect microbial activity in contrast to plant-derived material typically showing C/*N* ≥ 20 (Meyers, 1994), owing to the abundance of cellulose in vascular plants and its absence in microorganisms, together with the remineralization of carbon and immobilization of nitrogenous material by microbes (Sollins *et al.*, 1984; Schubert and Calvert, 2001). On the other hand, immunodetections of structural proteins involved in cell division and septation such as FtsZ were also generally detected throughout the M drill, except for the most surficial layer (Fig. 9a). The DNA sequencing indicated that the ubiquitous microbiology was dominated by members of the Actinobacteria, Bacilli, Clostridia, and Gammaproteobacteria classes (Fig. 5).

Microbial biomass signals showed certain distribution patterns with depth along the M drill. For instance, microorganisms commonly found in soil and decaying organic matter (Actinomycetales) were more abundantly measured down to 60-cm depth, along with Staphylococcaceae and spore-forming unclassified Clostridiales (Fig. 6a), both (facultative and strict, respectively) anaerobes. This was consistent with the immunodetection of spore-forming Firmicutes (*Bacillus*) and Actinobacteria (*Streptomyces*) with LDChip, mostly down to 60-cm depth (Fig. 9a). At the same time, the immunodetection of proteins involved in mechanisms of resistance to desiccation (SodA, sodF, and WshR) revealed a hydric stress in the deeper half of the M profile, more or less mirroring the distribution of proteins involved in reducing damage caused by oxidative stress (Ccda, PhcA2, DhnA2, and PufM2). Archaeal biomarkers, measured as both lipid proxies of halophilic archaea (*i.e*., squalane; ten Haven *et al.*, 1988) and positive immunodetections against *Halorubrum* spp. and *Pyroccoccus* spp., were preferentially found in the upper M layers (Figs. 4f and 8a, respectively).

In addition, microbes particularly resistant to heavy metals such as Moraxellaceae were also more abundant in upper layers (Fig. 6a), coinciding with immunodetection of Gammaproteobacteria (including Moraxellaceae) involved in sulfur oxidation (*i.e*., *Acidithiobacillus* spp, *Psicrobacter* sp., and *Halothiobacillus* sp.) (Fig. 9a). In contrast, heterotrophic Vibronaceae, also resistant to heavy metals, facultative anaerobes capable of doing fermentation, were relatively more abundant in samples below 60 cm, similar to Oceanospirillaceae (Fig. 6a).

Lipid biomarkers such as C_15_ and C_17_ *i-/a-* fatty acids related with sulfate-reducing bacteria (Kaneda, 1991) (Fig. 4b) or dicarboxylic acids (Fig. 4c) elsewhere described as proxies of thermophiles (Carballeira *et al.*, 1997) were increasingly detected with depth (Fig. 4b, c). Other source-specific lipid proxies such as the mono-unsaturated C_18:1_ (ω8) fatty acid (methanotrophic biosignatures; Boschker and Middleburg, 2002 and references therein) did not show a particular depth pattern (Fig. 4e).

Despite the acidic pH conditions (Fig. 1b), low but generalized presence of cyanobacterial markers was consistently detected by DNA sequencing (ca. 1%), LDChip (*e.g*., *Anabaena* spp.), and lipid biomarkers (C_18:1_ [ω9], and C_18:2_ [ω6] fatty acids; Cohen *et al.*, 1995; Allen *et al.*, 2010) along the M drill. So far, Cyanobacteria have not been found in acidophilic environments. Some of us have recently reported viable cyanobacteria in rocky cores from a 607m-depth borehole 500 m far northeast from our sampling site (IPBSL-2015 in Fig. 1), but at pH values higher than 6 (Puente-Sánchez *et al.*, 2018). The unexpected presence of these endolithic and hypolithic cyanobacteria in the dark deep subsurface of the Río Tinto area was explained by specific adaptations to environmental and nutritional stress, such as the use of molecular hydrogen as energy source (Puente-Sánchez *et al.*, 2018). This possibility can also be considered in this study, where Cyanobacteria could occupy specific high pH (≥4–5) microniches, which is not unreasonable considering the mineralogical (Fig. 2b, c) and geochemical (Fig. 3a) variability observed in barely 2 m of horizontal distance between the M and G drills. Also, inputs of allochthone organic matter from upper, surrounding less acid terrains would provide a suitable habitat for Cyanobacteria, such as those northward of the LMAP esplanade (*e.g.,* Puente-Sánchez *et al.*, 2018). In addition, the generalized presence of impermeable clay-rich and shale layers together with the hydric stress inferred from LDChip signals of hydric stress proteins (Fig. 9a) could contribute toward creating those high pH microniches for cyanobacterial strains.

This microbial community observed is consistent with a biomass δ^13^C and δ^15^N composition (Fig. 3g, h) reflecting carbon fixation pathways related to the Calvin cycle or reductive acetyl-CoA pathway (Hayes, 2001), and nitrogen metabolisms linked to N_2_ fixation via nitrogenase, organic N assimilation, and/or NH_4_^+^ production from organic matter decomposition (δ^15^N values from 0‰ to 6‰; Robinson, 2001). Microorganisms fixing carbon through the Calvin cycle (*e.g.,* Cyanobacteria, other photosynthetic bacteria, and some archaea) produce biomass δ^13^C values relative to CO_2_ ranging from −18‰ to −30‰ (Guy *et al.*, 1993), whereas those using the reductive acetyl-CoA pathway (variety of anaerobic, nonphotosynthetic bacteria and archaea) result in δ^13^C values from −23‰ to −44‰ (Preuβ *et al.*, 1989). In our drilled samples, different members of the Firmicutes, Alpha, Beta, and Gammaproteobacteria phyla are potential users of either of the two carbon fixation pathways; whereas members of Cyanobacteria and Alphaproteobacteria (Rhizobiales), as well as algae and/or plants are capable of nitrogen fixation.

Compared with the M drill samples, the microbial community in G was rather dominated by families involved in methylotrophic metabolism (Methylobacteriacea), acidophilic microorganisms that use O_2_ and Fe^+3^ as electron acceptors (Acetobacteraceae), heavy metal-resistant microorganisms (Rhodocyclaceae, Moraxellacea, and Enterobacteriaceae), or nitrite reducers (Xanthomonadaceae) (Fig. 6b). In contrast to the M community, no particular depth pattern was observed in the distribution of the microbial community along the G drill.

Overall, the difference between the M and G holes was remarkable for the lipid (Fig. 4), DNA (Figs. 5 and 6), and antibody biomarkers (Fig. 9). The microbial community in M was, overall, richer and less diverse than that along the G drill (Fig. 8). The depth pattern described earlier for the microbial community in M appeared to be somehow influenced by mineralogical and geochemical features, as the subsurface samples of the robotic drill were clustered in the CA according to their depth (Fig. 7). On the one hand, muscovite and acidic sulfates together with the biomass δ^13^C and chloride content seemed to be major drivers of the microbial community at the upper layers, largely represented by members of the Actinobacteria and Clostridia classes. On the other hand, the presence of pyrite was a key factor for explaining the microbial composition of the deeper communities in M, dominated by Gammaproteobacteria. In contrast, the microbial community in G, mostly represented by Alphaproteobacteria, did not follow a clear depth pattern (Fig. 7). Yet, certain differentiation was observed between deep samples (from 75 to 188 cm) largely influenced by the amount of goethite, formate, or TOC, and those from upper layers (from 35 to 60 cm depth) more affected by factors such as the proportion of quartz or the δ^15^N ratio (Fig. 7a). In summary, we postulate that the mineralogy and bulk geochemistry influenced habitability in this particular Río Tinto shallow subsurface Mars analog, where a number of biomarkers displayed distribution patterns along the depth profiles.

### 4.2. Different factors influencing the multianalytical microbial detection

The present multianalytical study produced some diverging results responding to different factors that deserve a detailed discussion according to their nature. Overall, result discrepancies were attributable to (i) different detection techniques (*i.e*., analysis of lipid material, DNA sequencing, or immunoassays), (ii) different sampling systems (*i.e*., robotic drill versus manual vibro-coring), and (iii) spatial variability of mineralogy and geochemistry.

The first factor attributes such discrepancies to the analytical constraints of each technique. On one hand, each of the three techniques applied here for detecting biomarkers has targets of different composition (*i.e*., free organic-soluble lipids versus DNA sequences versus diverse antigens), biological specificity, and resistance to degradation. Lipids are structural components of cell membranes, ubiquitous in all organisms and, thus, of less taxonomic specificity. As a counterpart, they are among the most recalcitrant organic biomarkers, with hydrocarbon skeletons being capable of resisting degradation for up to billions of years (Brocks and Summons, 2003). In contrast, genetic (*i.e*., nucleic acid sequences of DNA) and immunological (*i.e*., peptidic and nonpeptidic epitopes from microorganisms and biological polymers pursued by the LDChip) biomarkers are among the most labile biomolecules. The differential specificity and preservation potential of lipids, DNA, or antigens influence the detection capacity of each approach, thus leading us to expect results that are not necessarily comparable from a quantitative and even qualitative perspective. This explains the different level of taxonomic detail obtained from the lipids (detecting only certain general groups) versus the DNA or LDChip (detecting phylogenetic groups from phyla to family) results.

In addition, some biomarkers (lipids) show lower source specificity than others (DNA or antibodies) because of their nature (*i.e*., structural components of cell membranes generally present in most phylogenetic groups), thus offering a relatively less exclusive source diagnosis. That is the case with, for instance, squalane, dicarboxylic acids, or different polyunsaturated C_18_ fatty acids that, despite occurring potentially in other organisms even in most (*e.g.*, squalane), are likely to derive mostly from archaea (squalane; Brocks and Summons, 2003), cyanobacteria (C_18:1_ [ω9] and C_18:2_ [ω6]; Willers *et al.*, 2015 and references therein), or simply have been described just in certain groups to date (*e.g*., dicarboxylic acids on thermophiles, Carballeira *et al.*, 1997; or C_18:1_ [ω8] on methanotrophs, *e.g*., Bowman *et al.*, 1991 or Bodelier *et al.*, 2009). All this may explain, to some extent, the lack of agreement in the detection of certain groups (*e.g.*, archaea) by DNA sequencing, LDChip, and lipid biomarkers. Besides, a low abundance of archaeal biomass relative to other biological sources (*e.g*., bacteria, algae or plants) reduces the detection capability of its DNA by sequencing techniques. In contrast, the higher sensitivity of the immunological LDChip or the greater resistance to degradation of lipid remnants allows the detection of archaeal biomass even at low concentrations (Figs. 4f and 9).

Beyond being a limiting factor, handling such heterogeneous results has a positive aspect, that is, the complementary information the three biomarker types provide on biological terms, with DNA affording high taxonomic specificity from resisting genetic material, the LDChip providing high sensitivity on the detection of a defined number of microorganisms and proteins (*i.e*., 200 antibodies), and the lipid biomarkers offering the best estimation of the existing biomass with distinction of general sources (*i.e*., prokaryotic vs. eukaryotic, aquatic vs. terrestrial, or a number of source-specific microbes). On the other hand, each detection approach belongs to a different type of analytical method, where the LDChip is a microarray immunoassay interrogating a panel with a finite number of polyclonal antibodies (*i.e*., close method); whereas the other two approaches are able to detect any existing genetic (DNA) or structural (lipid) material (*i.e*., open methods). This difference explains the lack of detection by the LDChip of certain microbial groups that were detected by DNA sequencing (*e.g*., Alpha- and Betaproteobacteria, or Deinoccoci) in the M drill.

Finally, the size of analytical sample varies considerably from one to another approach (*i.e*., 10–25 g of subsurface sample for lipid extraction, 10 g for DNA extraction, and 0.5 g for the LDChip immunoassay), which certainly affected how representative the analytical sample is of the whole field sample (*i.e*., material from each depth interval).

The second factor refers to the different approach followed to collect samples from the Río Tinto-analog subsurface. On one hand, the Trident drill autonomously accessed the terrain to 100-cm depth for acquiring small subsurface samples (*i.e*., ≤70 g) in the form of cuttings from 20-cm-depth intervals. On the other hand, about 100–200 g of subsurface material was sampled manually at different depth intervals (covering different visually determined color layers; Fig. 2a) from the 200-cm-deep G drill acquired with a manual vibro-corer. The different (collection) sample size and slightly different depth interval covered by each sampling technique, as well as the mixing (M) versus not-mixing (G) procedures, may explain part of the divergences observed between the biomarker patterns in the M versus G profiles, such as those of lipid proxies (*e.g.*, archaeal squalane; Fig. 4f), phylogenetic groups (*e.g.*, Proteobacteria or Cyanobacteria; Figs. 5 and 9), or metabolic processes (Fig. 9).

Complementary with the latter, a third and last diverging factor is the spatial geochemical and mineralogical heterogeneity across the sampled terrain that may also contribute to explaining the different biomarker profiles between the M and G drills. In about 2 m of distance, mineralogical (*e.g*., pyrite and hexahydrite; Fig. 1b, c) and geochemical features such as the concentration of sulfate, formate, or acetate (Fig. 3a, d, and e, respectively) varied considerably from the M to the G drills. Small-scale heterogeneity is an important factor for being considered in any drilling analysis both on Earth and on Mars, with sample homogenization being a difficulty in robotic missions. In fact, in a comparable distance (*i.e.*, ∼140 cm), differences in the bulk geochemistry (calcium oxide and calcium sulfates) were observed between the Cumberland and John Klein holes, the first two martian drills analyzed by the Mars Science Laboratory in the apparently uniform sedimentary Sheepbed Mudstone, an ancient lacustrine environment in Yellowknife Bay (Jackson *et al.*, 2016).

The spatial variability is a relevant aspect to be taken into account in life detection missions, as it likely plays a role in defining environmental differences at a microscale (*i.e*., niches) that certainly influences the preferential development of certain microorganisms or the differential preservation of their remnants. Consequently, small-scale heterogeneity may have an important impact on the drilling and sampling strategies in missions such as *IceBreaker* (McKay *et al.*, 2013).

Altogether, factors related to analytical constraints, biological specificity, chemical resistance, sampling approach, and environmental heterogeneity contributed to producing heterogeneous results hardly comparable from a quantitative perspective, but rather complementary in the forensic information they supply from biomarkers of different scope (lipids, DNA sequences, or immunodetections).

## 5. Conclusions

The autonomous lander-mounted drilling system successfully demonstrated fully robotic and intelligent sample acquisition and transfer for biomarker detection in the Río Tinto-Mars analog site. The Trident drill mounted on a NASA Phoenix/InSight-like mockup platform provided subsurface samples that allowed for a multianalytical investigation of microbial diversity at a high level of detail (structural, phylogenetic, and metabolic). The parallel manual sampling and analysis of lipid, genetic, and immunological biomarkers demonstrated feasibility and fidelity of the automated biomarker-searching approach. A diverse subsurface microbial community was found to be ubiquitous, with a certain association of microbial biomarkers with abiotic variables such as mineralogy, bulk geochemistry, and/or local scarcity of water.

The spatial heterogeneity of such abiotic variables at a local microscale, thus, raised the relevance of considering several drilling sites for achieving a good coverage and local representativity of the target area. Still, sampling different lithologies may be also paramount for increasing the chances of detecting life biomarkers. Assuming the complexity of deciding on the best sampling targets and considering the limited number of drills that could be taken in a given life detection mission, a compromise must be reached between lithological variability and spatial coverage of a priority target area. This is an important aspect for future Mars astrobiology missions, where the window of success is narrow and detecting low concentrations (if any) of potential biosignatures is complicated.

Detecting residual biosignatures in extreme environments such as the martian subsurface may prove highly challenging owing to the combination of expected low biomass, existence of mineral and rocky refugia in a variable mineralogy and geochemistry, and a resulting patchy biomarker distribution. Assuming that any potential biomarker on Mars is expected to be scarce and diffusedly scattered throughout the martian subsurface (even forming micro-niches), covering the maximum extension possible (both in depth and horizontal distance) is key for increasing the chances of detection.

Aiming for the best radiation-preserved (perhaps organic) material, future ESA ExoMars rover mission plans to drill down to 2 m, and the *IceBreaker* mission concept plans to go down to 1m depth. We report here a successful demonstration of autonomous drilling down to 1m depth as a proof-of-concept study. The drill apparatus employed in this study has demonstrated that subsurface biosignatures spanning a wide range of compositional nature, preservation potential, and taxonomic specificity can be recovered from an iron-rich Mars analog site. Although genetic biosignatures (*i.e*., DNA sequences) may not be the primary method used to search for traces of life on Mars, their use in this study corroborated and complemented the information obtained from other methods currently used or foreseen in future missions (*i.e*., lipid GC-MS, and SOLID-LDChip). The high preservation potential of lipid biomarkers, the high sensitivity of the LDChip for detecting biopolymers, and the complementary information provided by both techniques make them fundamental components for *IceBreaker* mission payload for searching for molecular evidence of life in the martian ice-cemented subsurface.

## Abbreviations Used

ACL: average chain length
ARADS: Atacama Rover Astrobiology Drilling System
BSTFA: N,O-bis[trimethylsilyl]trifuoroacetamide
CA: correspondence analysis
CPI: carbon preference index
dw: dry weight
FAMEs: fatty acid methyl esters
FSMI: fluorescent sandwich microarray immunoassay
GC-MS: gas chromatography–mass spectrometry
HMW: high molecular weight
IC: ion chromatography
IPB: Iberian Pyritic Belt
IRMS: isotope-ratio mass spectrometry
LDChip: life detector chip
LMAP: Life-detection Mars Analog Project
LMW: low molecular weight
n.d.: not detected
OTUs: operational taxonomic units
PCR: polymerase chain reaction
SOLID: Signs of LIfe Detector
TLE: total lipid extracts
TN: total nitrogen
TOC: total organic carbon
